# Polyphenol-Rich Snack Consumption during Endurance Exercise Training Improves Nitric Oxide Bioavailability but does not Improve Exercise Performance in Male Cyclists: A Randomised Controlled Trial

**DOI:** 10.1016/j.cdnut.2025.106006

**Published:** 2025-03-24

**Authors:** Noah Marc Adrian d’Unienville, Alison M Coates, Alison M Hill, Maximillian J Nelson, Kevin Croft, Catherine Yandell, Jonathan D Buckley

**Affiliations:** 1 Alliance for Research in Exercise, Nutrition and Activity (ARENA), UniSA Allied Health and Human Performance, University of South Australia, Adelaide, Australia; 2 UniSA Clinical and Health Sciences, University of South Australia, Adelaide, Australia; 3 School of Biomedical Sciences, Pharmacology and Toxicology, University of Western Australia, Perth, Australia

**Keywords:** cycling performance, recovery, sports nutrition, functional foods, athletes

## Abstract

**Background:**

Antioxidants and nitric oxide (NO) precursors may improve endurance exercise performance by reducing oxidative stress and increasing NO production. Almonds, dried grapes, and cranberries (AGC) are good sources of antioxidants and NO precursors.

**Objectives:**

To determine whether AGC consumption improved physiological responses and endurance cycling time-trial performance in response to training.

**Methods:**

After 1 wk of light training (LT), 96 male recreationally trained cyclists consumed 125 g of AGC or control (CON: isocaloric oat bar) daily during 2 wk of heavy training (HT) and a 2-wk taper (T). At the end of LT, HT, and T, endurance exercise performance (5-min cycling time-trial; 5CTT), NO bioavailability (plasma and urine nitrate and nitrite), oxidative stress [plasma F2-isoprostanes (F_2_-Isop)], muscle damage (creatine kinase) and subjective measures of wellbeing were assessed, as well as physiological responses during exercise at 70% maximal aerobic power output.

**Results:**

Compared to LT, 5CTT performance was impaired at HT (*d* = –0.27, *P* = 0.01) and improved at T (*d* = 0.79, *P* < 0.001), with no difference between treatments (*P* > 0.81). Compared with CON, during submaximal exercise at 70%, maximal aerobic power output AGC demonstrated higher oxygen consumption (HT: *d* = 0.46; T: *d* = 0.38, *P* < 0.001) and lower respiratory exchange ratio (HT: *d* = –0.61; T: *d* = –0.23, *P* < 0.032). At HT, urine F_2_-Isop was higher compared with LT (*d* = 0.21, *P* = 0.036), but plasma F_2_-Isop was lower (*d* = –0.22, *P* = 0.008*)*, with no difference between treatments. At HT, AGC had higher subjective energy concentrations (*d* = 0.21, *P* = 0.02) and urinary nitrite (*d* = 0.23, *P* = 0.03) compared with CON and higher creatine kinase (*d* = 0.24, *P* = 0.02) and less fatigue (*d* = –0.20; *P* = 0.05) at T.

**Conclusions:**

Although not beneficial for 5CTT performance or exercise efficiency, AGC increases fat oxidation during exercise, NO bioavailability, and subjective energy concentrations, which may confer benefits for health and wellbeing.

This trial was registered at www.anzctr.org.au as ACTRN12618000360213.

## Introduction

Strenuous exercise generates reactive oxygen species (ROS), which are important for influencing both acute responses and chronic adaptations to exercise [1]. Acutely, high levels of ROS may induce cellular damage and promote fatigue during exercise by impairing muscular blood flow [2], impairing muscle excitation-contraction coupling through altered calcium handling at the sarcoplasmic reticulum and decreased myofibrillar calcium sensitivity [1,3], reducing central nervous system drive [4] and contributing to muscle damage and inflammation [4]. Thus, interventions that can limit oxidative stress caused by ROS during exercise may be beneficial for exercise performance.

Dietary polyphenols are bioactive phytochemicals distinguished by multiple phenolic rings [5]. Polyphenols possess potent antioxidant properties, and their consumption has been proposed to enhance exercise performance and recovery by mitigating imbalances between ROS and antioxidants (i.e., reducing oxidative stress) [4] and increasing the bioavailability of nitric oxide (NO) [6]. NO is a signaling molecule that has demonstrated the capacity to promote vasodilatation [7] and enhance skeletal muscle contractility [8] in humans. NO is synthesized from L-arginine and oxygen in a reaction catalyzed by various NO synthase enzymes, including endothelial NO synthase. Polyphenols can increase NO bioavailability both by enhancing endothelial NO synthase expression and protecting NO from breakdown by ROS [3,6]. Increasing NO bioavailability may enhance muscle perfusion and improve mitochondrial function to reduce the oxygen cost of exercise, also contributing to improved endurance exercise performance [9]. However, although consumption of polyphenols has broadly demonstrated minor benefits for endurance exercise performance, there is considerable heterogeneity in physiological and performance effects attributable to variations in food sources, food matrices (e.g., whole foods compared with extracts) and population characteristics (e.g., sex, fitness level) [10].

Almonds, dried grapes (e.g., raisins, sultanas), and dried cranberries contain high amounts of polyphenols [11], with total phenolic contents of 287 mg, 1065 mg, and 443 mg (gallic acid equivalents) per 100 g, respectively [12,13], whereas almonds are also a source of monounsaturated fats, antioxidants such as α-tocopherol (vitamin E), and L-arginine [14], a precursor for NO. Thus, consumption of almonds, grapes, and cranberries (AGC) has the potential to not only increase NO synthesis through increased availability of L-arginine but also increase antioxidant activity against ROS, thereby reducing oxidative stress [11] and promoting NO bioavailability.

This study evaluated the effects of consuming AGC on exercise-induced oxidative stress, muscle damage, subjective recovery, NO bioavailability, submaximal oxygen consumption, substrate utilization, and maximal endurance exercise performance in male recreationally trained cyclists. Male cyclists were chosen because females have greater endothelium-dependent dilatation [15] and may have enhanced antioxidant mechanisms (e.g., increased concentrations of antioxidant enzymes) and reduced levels of oxidative stress compared with males [16], so foods rich in polyphenols and NO precursors may confer greater benefits for males than females.

The primary hypotheses tested in the present study were that compared to a low-polyphenol isocaloric control (CON), AGC consumption would *1*) reduce the decline in endurance exercise performance after intense training and *2*) further improve endurance exercise performance following a taper period. Secondary hypotheses were that relative to CON, consumption of AGC would *1*) reduce markers of exercise-induced oxidative stress and muscle damage, *2*) improve subjective markers of recovery, *3*) increase markers of NO bioavailability, and *4*) improve submaximal exercise efficiency (via decreased oxygen consumption). Exploratory hypotheses were that *1*) the decline in performance following intense training would be inversely related to changes in markers of oxidative stress and muscle damage, and *2*) improvements in exercise efficiency and maximal exercise performance would be positively associated with changes in NO bioavailability.

## Methods

This single-blind trial was approved by the University of South Australia Human Research Ethics Committee (approval # 200372), with outcome assessors blinded to the treatment allocation of participants. A detailed study protocol has been published previously [17], and this study presents key findings related to exercise performance.

Yi et al. [18] reported a near-significant 5.3% improvement in cycling time-trial performance (effect size of 0.58) compared with placebo following 4 wk of consuming 75 g/d of almonds. Based on the mean and SD of 5-min cycling time trial (5CTT) performance in our laboratory’s previous studies using the same testing and training methodologies [19,20], replicating this effect size with statistical significance in a 2-tailed test with 80% power and at an α-level of 0.05 required 96 participants (1:1 allocation).

To summarize this study’s methodology, male recreationally trained cyclists who had been training ≥3 times per week for 6 mo were assessed after a 1-wk run-in period of light training (LT), after 2 wk of heavy training (HT: ∼2 h/d of interval training) designed to induce substantial fatigue and impair performance, and 2 wk of tapered training (T) intended to enable physiological adaptations to occur to improve endurance exercise performance. LT included daily training sessions of 30–60 min at 65–85% max heart rate (HR), whereas each day of HT comprised 16 min of warmup/cool down and 4 repeats of 8.5 min at zone 2 (69–81% max HR), 8 min at zone 3 (82–87% max HR), 7.5 min at zone 4 (88–94% max HR), and 3 min at zone 5 (≥95% max HR). T included 3 rest days, a mix of training sessions of 50–60 min at 65–85% max HR, and a single 30-min-long interval training session (6 repeats of 3 min at zone 2 and 3 min at zone 4). HR and rating of perceived exertion (RPE) were recorded during all training sessions to assess compliance and compare training loads between groups. A researcher not involved in recruitment or data collection randomly allocated participants via minimization [using 5CTT kJ/kg, age, and BMI (in kg/m^2^)] to consume 2550 kJ of AGC, comprising almonds (75 g), dried grapes (i.e., sultanas; 25 g) and dried cranberries (25 g) or an energy-matched oat bar (CON: ∼132 g) each day during the 5 wk of training. Compliance with experimental food consumption was confirmed through a daily checklist, and any unconsumed treatment foods were returned. Further details on dietary and training compliance are available in the protocol article [17]. Three-day weighed food records were also used during each training period to monitor intakes of energy and key nutrients (Foodworks Professional Software version 8; Xyris Software Inc). Participants were provided with a list of food sources that were rich in polyphenols (e.g., specific fruits, coffee, tea, and chocolate) and nitrate (e.g., beetroot and green leafy vegetables) and were asked to avoid consuming these foods in large quantities or high concentrations (e.g., from supplements). Participants also recorded their daily intakes of nitrate- or polyphenol-rich foods to permit estimation of nitrate and polyphenol consumption using reference values from Rothwell et al. [21] and Blekkenhorst et al. [22], respectively.

Participants reported to the laboratory for testing following an overnight fast on the days following the end of LT, HT, and T, and provided 24-h urine collections and had a venous blood sample collected. Urine and plasma F_2_-isoprostanes (F_2_-Isop) were analyzed to evaluate oxidative stress, with reported coefficients of variation (CV) of 3.7% and 5.6%, respectively, when analyzed using our method [23]. Plasma and urinary nitrite and nitrate concentrations, which typically have a CV of <8% [[24], [25], [26], [27]], were assessed as indicators of NO bioavailability. Although there is some conjecture in the literature regarding whether serum creatine kinase (CK) is an accurate marker of muscle damage or more a reflection of altered muscle cellular metabolism during light-to-moderate intensity exercise, it is more widely accepted as a marker of muscle damage in response to high-intensity sustained exercise [28]. Therefore, it was included as a marker of muscle damage in the present study, given the high intensity and prolonged duration of training during HT. The reported day-to-day coefficient of variation for serum CK is 19% [29]. Subjective recovery was assessed via a paper-based modified Daily Analysis of Life Demands for Athletes questionnaire [30], which tracked the number of “Worse than normal” responses and changes in perceived mood, energy, stress, soreness, and fatigue. The Daily Analysis of Life Demands for Athletes questionnaire has demonstrated strong associations with changes in training load and athletic performance [31] and has shown to be responsive to the training intervention used in the current study [19].

At each testing session, after a 5-min warmup, participants began a sustained exertion test (SET), which involved cycling at 70% of maximal aerobic power for 45 min. Five minutes of expired gases were collected at the 10-min, 25-min, and 40-min timepoints of this test. Upon completion of the SET, they rested for 15 min before completing a 5CTT as a measure of maximal endurance exercise performance, which has demonstrated a CV of 1.2% in our laboratory [32].

Data are presented as mean ± (SD), and the magnitude of effects are reported as Cohen’s *d* with effect sizes interpreted as follows: <0.20 = trivial, 0.20 = small, 0.5 = medium, and 0.80 = large [33]. Blinded statistical analysis was performed using Stata/IC 16.1 (StataCorp LLC) using linear mixed-effects models, with fixed effects entered as outcome measures, treatment allocation and timepoint, and participant ID entered as a random effect. Residuals were analyzed to ensure homoscedasticity. In the absence of significant treatment by timepoint interaction effects, timepoints were compared pairwise. *P* values for the false discovery rate as per Benjamini et al. [34]. Covariates included age, training compliance, and dietary intervention compliance. Regression analyses evaluated associations between changes in CK and F_2_-Isop in plasma and urine and between all biochemical measures and 5CTT performance changes. Where participant data for a timepoint were unavailable due to technical issues or dropout, their missing data were imputed using maximum likelihood estimation and included in the analysis provided data were missing at random. Statistical significance was set at an α-level of 0.05.

## Results

**FIGURE 1:**
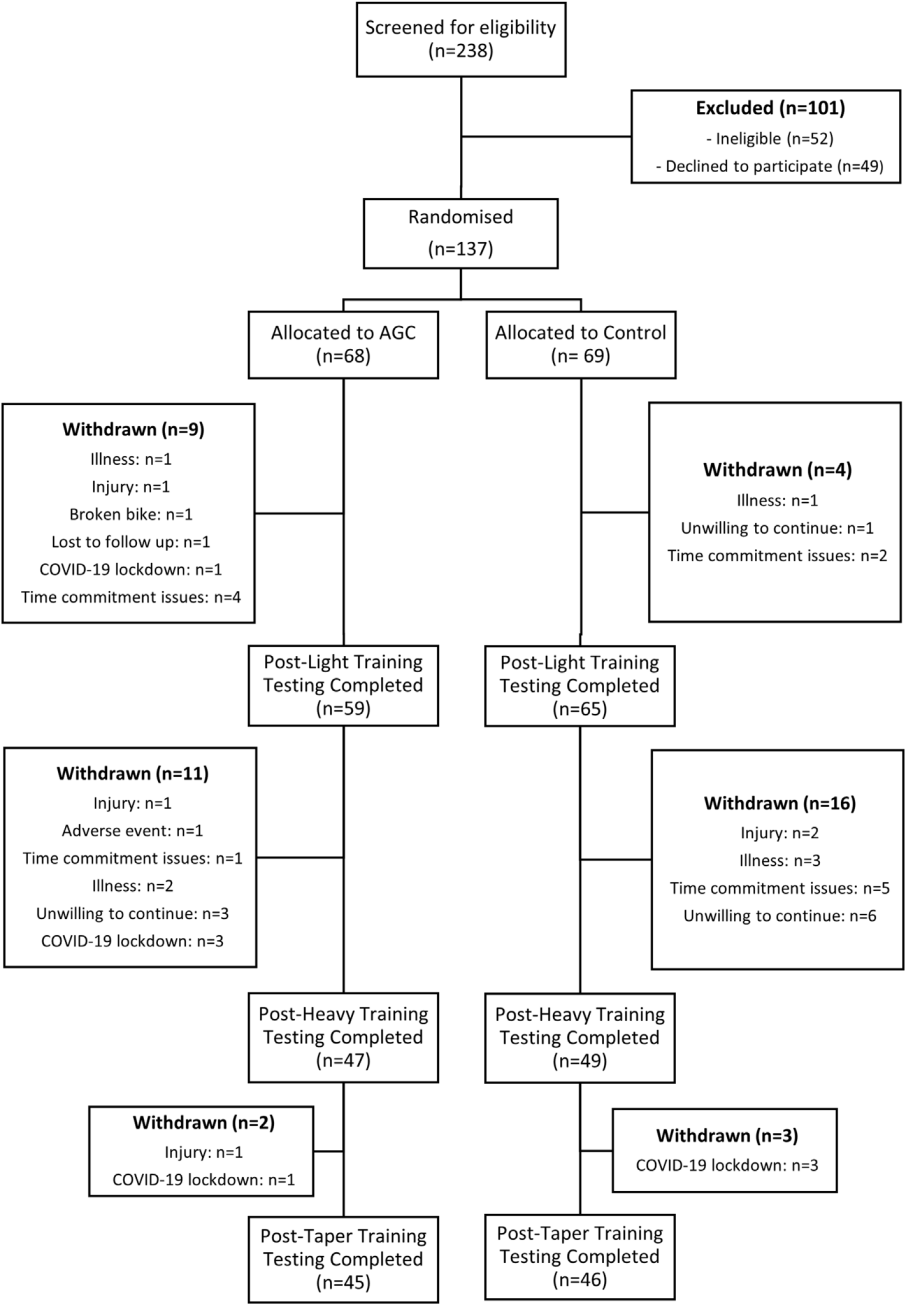
Participant flow diagram. AGC, almonds, dried grapes, and cranberries; COVID-19, coronavirus disease 2019.

One hundred thirty-seven participants were randomized, and 91 completed all stages of the study (Figure 1) between March 2018 and April 2021. There were no differences in randomization variables (age, BMI, and 5CTT) for participants who completed LT and HT and those who completed all stages of the study, suggesting that data for participants who completed LT and HT (*n* = 96) but not T were missing at random, and all available data for these participants were therefore included in the analysis. Baseline 5CTT performance in participants who completed only LT was lower than those who completed all stages of the study, and their results were therefore not considered to be missing at random and were not included in the analyses. Data from 6 participants (3 in each treatment arm) who completed the study were excluded due to not meeting the HT training compliance requirements of reporting a mean RPE of ≥15 for each training session; thus, data analyses were based on 90 participants (AGC *n* = 44; age yr: 42.6 ± 10.5; BMI kg/m^2^: 24.7 ± 2.6; 5CTT kJ/kg: 1.19 ± 0.18; CON *n* = 46; age yr: 41.8 ± 10.7; BMI kg/m^2^: 24.7 ± 2.7; 5CTT kJ/kg: 1.19 ± 0.18). Exercise data were missing from 4 of these participants at HT (2 due to inability to attend, 2 due to acute pain), 2 of which could not provide blood plasma/serum samples, and 1 participant’s 5CTT total work done was missing due to recording error. No adverse events were reported in response to consuming the intervention foods.

### Dietary intake and diet treatment compliance

Dietary intake data are provided in Table 1. Daily energy intake was increased in both AGC and CON from LT to HT and remained elevated at T (*P* < 0.001). From LT to HT, compared to CON, AGC had small to moderate increases in daily consumption of total fat, monounsaturated fat, polyunsaturated fat, total dietary polyphenols, a large increase in α-tocopherol intake, and reduced percentages of energy intake from carbohydrate and saturated fat. These differences were maintained at T. There were no between-group changes in the consumption of dietary polyphenols and nitrates in the background diet, but there were small increases in overall polyphenol consumption at HT and T in AGC compared with CON due to the consumption of the study foods (*P* < 0.001).

**TABLE 1:** Mean daily dietary intakes.

	LT	HT	T	LT to HT	LT to T
Energy (kJ)					
AGC	11,940 ± 3591	14,819 ± 3467	14,473 ± 3167	Time: d = 0.74, P < 0.001∗	Time: d = 0.63, P < 0.001∗
Control	12,411 ± 3005	14,262 ± 3071	14,241 ± 2479	T × T: d = 0.19, P = 0.074	T x T: d = 0.09, P = 0.382
Protein (g)					
AGC	126 ± 47	144 ± 42	142 ± 40	Time: d = 0.39, P = 0.001∗	Time: d = 0.37, P = 0.002∗
Control	126 ± 31	138 ± 40	140 ± 25	T × T: d = 0.07, P = 0.496	T x T: d = 0.02, P = 0.859
% Energy from protein					
AGC	18 ± 4	17 ± 3	17 ± 3	Time: d = -0.37, P = 0.01∗	Time: d = –0.26, P = 0.001∗
Control	18 ± 4	17 ± 3	17 ± 3	T × T: d = -0.08, P = 0.47	T x T: d = –0.08, P = 0.464
Total fat (g)					
AGC	106 ± 41	156 ± 44	153 ± 43	Time: d = 0.88, P < 0.001∗	Time: d = 0.85, P < 0.001∗
Control	108 ± 36	125 ± 29	130 ± 24	T × T: d = 0.52, P < 0.001∗	T x T: d = 0.35, P = 0.001∗
% Energy from fat					
AGC	32 ± 6	39 ± 4	39 ± 5	Time: d = 0.60, P < 0.001∗	Time: d = 0.70, P < 0.001∗
Control	32 ± 7	33 ± 5	34 ± 4	T × T: d = 0.57, P < 0.001∗	T x T: d = 0.46, P < 0.001∗
Saturated fat (g)					
AGC	40 ± 18	48 ± 23	47 ± 21	Time: d = 0.52, P < 0.001∗	Time: d = 0.48, P < 0.001∗
Control	41 ± 15	51 ± 14	53 ± 10	T × T: d = –0.09, P = 0.398	T x T: d = –0.21, P = 0.045∗
% Energy from SFA					
AGC	12.2 ± 3.5	11.6 ± 3	11.6 ± 2.8	Time: d = 0.12, P = 0.195	Time: d = 0.16, P = 0.101
Control	12.2 ± 3.7	13.4 ± 3.1	13.9 ± 2.7	T × T: d = –0.30, P = 0.004∗	T x T: d = –0.37, P < 0.001∗
Monounsaturated fat (g)					
AGC	40 ± 16	69 ± 15	69 ± 17	Time: d = 1.07, P < 0.001∗	Time: d = 1.08, P < 0.001∗
Control	42 ± 16	45 ± 12	48 ± 12	T × T: d = 0.87, P < 0.001∗	T x T: d = 0.77, P < 0.001∗
% Energy from MUFA					
AGC	12.1 ± 2.8	17.4 ± 2.4	17.8 ± 3	Time: d = 0.78, P < 0.001∗	Time: d = 0.86, P < 0.001∗
Control	12.5 ± 3.4	11.8 ± 2.4	12.1 ± 2.9	T × T: d = 0.95, P < 0.001∗	T x T: d = 0.94, P < 0.001∗
Polyunsaturated fat (g)					
AGC	17 ± 8	28 ± 9	26 ± 6	Time: d = 0.78, P < 0.001∗	Time: d = 0.75, P < 0.001∗
Control	17 ± 7	18 ± 7	20 ± 8	T × T: d = 0.67, P < 0.001∗	T x T: d = 0.39, P < 0.001∗
% Energy from PUFA					
AGC	5.2 ± 1.8	7.0 ± 1.6	6.7 ± 1.4	Time: d = 0.46, P < 0.001∗	Time: d = 0.48, P < 0.001∗
Control	5.0 ± 1.5	4.8 ± 1.3	5.0 ± 1.6	T × T: d = 0.64, p < 0.001∗	T x T: d = 0.46, P < 0.001∗
Carbohydrate (g)					
AGC	311 ± 98	357 ± 99	342 ± 82	Time: d = 0.54, P < 0.001∗	Time: d = 0.34, P < 0.001∗
Control	340 ± 111	404 ± 113	386 ± 102	T × T: d = –0.12, P = 0.255	T x T: d = –0.14, P = 0.176
% Energy from CHO					
AGC	43 ± 8	39 ± 6	39 ± 5	Time: d = –0.15, P < 0.095	Time: d = –0.33, P < 0.001∗
Control	45 ± 8	46 ± 6	44 ± 6	T × T: d = –0.48, P < 0.001∗	T x T: d = –0.38, P < 0.001∗
Fiber (g)					
AGC	37 ± 15	44 ± 13	44 ± 13	Time: d = 0.36, P < 0.001∗	Time: d = 0.30, P = 0.002∗
Control	41 ± 18	46 ± 20	46 ± 17	T × T: d = 0.07, P = 0.482	T x T: d = 0.03, P = 0.765
Alcohol (g)					
AGC	10.4 ± 13.9	8.3 ± 9.8	10.8 ± 13	Time: d = –0.23, P = 0.03∗	Time: d = 0.02, P = 0.821
Control	7.7 ± 12.4	6.2 ± 9.1	9.0 ± 11.0	T × T: d = –0.06, P = 0.596	T x T: d = –0.05, P = 0.626
α-tocopherol (mg)					
AGC	15 ± 6	37 ± 7	37 ± 6	Time: d = 1.84, P < 0.001∗	Time: d = 1.77, P < 0.001∗
Control	15 ± 5	15 ± 4	15 ± 5	T × T: d = 2.12, P < 0.001∗	T x T: d = 1.93, P < 0.001∗
Background dietary nitrate (mg)					
AGC	30 ± 35	26 ± 29	21 ± 26	Time: d = –0.05, P = 0.639	Time: d = –0.12, P = 0.248
Control	48 ± 69	44 ± 65	45 ± 72	T × T: d = 0.00, P = 0.996	T x T: d = –0.04, P = 0.714
Background dietary polyphenols^1^ (mg)					
AGC	1888 ± 1194	1711 ± 1152	1858 ± 1090	Time: d = –0.18, P = 0.009∗	Time: d = –0.15, P = 0.029∗
Control	1956 ± 1067	1763 ± 999	1681 ± 910	T × T: d = 0.01, P = 0.959	T x T: d = 0.18, P = 0.081
Total dietary polyphenols (mg)					
AGC	1888 ± 1194	2271 ± 1152	2418 ± 1090	Time: d = 0.08, P < 0.250	Time: d = 0.10, P < 0.134
Control	1956 ± 1067	1763 ± 999	1681 ± 910	T × T: d = 0.42, P < 0.001∗	T x T: d = 0.58, P < 0.001∗

Abbreviations: AGC, almonds, dried grapes, and cranberries; CHO, carbohydrate; *d*, Cohen’s *d* (i.e., standardized mean difference); HT, heavy training; LT, light training; MUFA, monounsaturated fatty acid; PUFA, polyunsaturated fatty acid; SFA, saturated fatty acid; T **×** T, treatment X time interaction for AGC compared to control; T, taper training.

^∗^Indicates *P* < 0.05.

1 Does not include polyphenols consumed as part of dietary intervention.

Compliance with consumption of the study foods, as assessed by foods returned, was 90.8 ± 7.7% for CON and 89.7 ± 8.8% for AGC, with no difference in the percentage of foods consumed between the groups overall or at HT or T.

### Training intervention compliance

There were no differences between groups in compliance with the training program or any measure of training load across LT, HT, or T (Table 2).

**TABLE 2:** Training compliance.

Overall training compliance					
	LT	HT	T	LT to HT	LT to T
Duration (minutes)					
AGC	268 ± 26	1517 ± 160	513 ± 59	T × T: P = 0.980	T × T: P = 0.852
Control	271 ± 36	1524 ± 156	520 ± 62		
TRIMP (AU)					
AGC	34003 ± 5190	205445 ± 26740	65696 ± 9657	T × T: P = 0.978	T × T: P = 0.906
Control	35520 ± 5142	206647 ± 24794	67241 ± 9962		
TRIMP compliance (%)					
AGC	109 ± 10	94 ± 11	103 ± 12	T × T: P = 0.851	T × T: P = 0.219
Control	110 ± 10	95 ± 10	108 ± 15		
Mean session RPE					
AGC	3.3 ± 0.9	8.6 ± 1.3	3.6 ± 1.0	T × T: P = 0.608	T × T: P = 0.239
Control	3.1 ± 0.9	8.2 ± 1.4	3.7 ± 1.1		
Training load (AU)					
AGC	864 ± 234	13117 ± 2496	1781 ± 520	T × T: P = 0.351	T × T: P = 0.618
Control	833 ± 274	12707 ± 2257	1979 ± 819		

Heavy training heart rate zone compliance (%)^1^								
	Zone 2		Zone 3		Zone 4		Zone 5	
AGC	125 ± 30	P = 0.490	102 ± 34	P = 0.937	48 ± 30	P = 0.998	21 ± 27	P = 0.593
Control	130 ± 37		102 ± 30		48 ± 29		19 ± 21	

Abbreviations: AGC, almonds, dried grapes, and cranberries; AU, arbitrary units; HT, heavy training; LT, light training; RPE, rating of perceived exertion; T, taper training; T × T, treatment × time interaction for AGC compared to control; TRIMP, training impulse

1 Proportion of completed compared with prescribed time spent in heart rate training zones.

### Body mass

At LT, body mass was 78.5 ± 9.2 kg in AGC and 77.8 ± 9.0 kg in CON. Body mass did not change by HT (AGC 77.8 ± 9.0 kg, CON 77.7 ± 9.2 kg), but by the end of T, there was a small reduction in CON compared with AGC (AGC 78.7 ± 9.2 kg, CON 77.5 ± 9.0 kg, *P* = 0.02).

### Markers of oxidative stress, muscle damage, and NO bioavailability

From LT to HT, urinary nitrite was reduced in CON compared to AGC (Table 3), although this difference did not remain significant at T (*P* = 0.062). From LT to HT, there was a decrease in plasma F_2_-Isop and an increase in urine F_2_-Isop, but these returned to LT levels by T and were not different between treatments. There were no changes in any other measures of nitrate or nitrite. Concentrations of CK were unchanged from LT at HT but were increased in AGC compared with CON at T. Across all timepoints, there were also borderline-significant relationships between CK and both plasma (β = 0.00009, *P* = 0.05) and urinary (β = 0.003, *P* = 0.05) F_2_-Isop.

**TABLE 3:** Markers of oxidative stress, muscle damage, and nitric oxide bioavailability.

	LT	HT	T	Baseline to HT	Baseline to taper
Plasma F_2_-Isop (pmol/L)					
AGC	626 ± 174	570 ± 172	597 ± 180	Time: d = –0.22, P = 0.008∗	Time: d = 0.03, P = 0.695
Control	567 ± 160	538 ± 154	598 ± 176	T × T: d = –0.07, P = 0.502	T × T: d = –0.20, P = 0.053
Urinary F_2_-Isop (pmol/L)					
AGC	5547 ± 2697	6311 ± 2950	6668 ± 3826	Time: d = 0.21, P = 0.036∗	Time: d = 0.08, P = 0.429
Control	5856 ± 3637	6212 ± 3479	5446 ± 2729	T × T: d = –0.01, P = 0.926	T × T: d = 0.13, P = 0.216
Plasma nitrite (μM)					
AGC	1.67 ± 1.04	1.58 ± 1.07	1.61 ± 1.22	Time: d = –0.18, P = 0.126	Time: d = –0.21, P = 0.079
Control	1.63 ± 0.68	1.53 ± 0.84	1.47 ± 0.57	T × T: d = 0.08, P = 0.458	T × T: d = 0.04, P = 0.677
Urinary nitrite (μM)					
AGC	100.6 ± 112.4	100.1 ± 88.2	102.9 ± 67.7	Time: d = –0.12, P = 0.295	Time: d = 0.02, P = 0.885
Control	92.1 ± 68.4	67.2 ± 57.4	78.1 ± 60.9	T × T: d = 0.23, P = 0.029∗	T × T: d = 0.20, P = 0.062
Plasma nitrate (μM)					
AGC	25.6 ± 13.6	26.9 ± 13.6	24.2 ± 13.8	Time: d = –0.07, P = 0.563	Time: d = –0.19, P = 0.099
Control	30.1 ± 16.8	29.5 ± 17.2	26.1 ± 14.2	T × T: d = 0.14, P = 0.179	T × T: d = 0.14, P = 0.199
Urinary nitrate (μM)					
AGC	581 ± 502	696 ± 457	565 ± 358	Time: d = 0.20, P = 0.085	Time: d = –0.11, P = 0.369
Control	658 ± 351	667 ± 334	608 ± 391	T × T: d = 0.16, P = 0.13	T × T: d = 0.10, P = 0.321
Creatine kinase (U/L)					
AGC	127 ± 47	151 ± 90	159 ± 93	Time: d = 0.21, P = 0.054	Time: d = 0.06, P = 0.557
Control	157 ± 102	171 ± 102	145 ± 73	T × T: d = 0.05, P = 0.661	T × T: d = 0.24, P = 0.021∗

Abbreviations: AGC, almonds, dried grapes, and cranberries; *d*, Cohen’s *d* (i.e., standardized mean difference); F2-Isop, F2-isoprostanes; HT, heavy training; LT, light training; T, taper training; T × T, treatment × time interaction for AGC compared to control.

∗*P* < 0.05.

### Time-trial performance

Relative (kilojoules per kilogram) 5CTT performance declined from LT to HT by 2.0 ± 7.4% (*P* < 0.001) but was 5.5 ± 7.4% higher than LT by the end of T (*P <* 0.001). The changes in absolute 5CTT performance followed a similar pattern, as shown in Table 4, with no differences between treatments for absolute or relative 5CTT performance.

**TABLE 4:** Exercise testing parameters.

	LT	HT	T	LT to HT	LT to taper
Five-minute time-trial					
Total work (kJ)					
AGC	96.8 ± 15.8	94.6 ± 14.6	102.4 ± 16.4	Time: d = –0.14, P < 0.001∗	Time: d = 0.36, P < 0.001∗
Control	95.9 ± 15.0	92.6 ± 14.1	101.0 ± 14.5	T × T: d = 0.01, P = 0.891	T × T: d = 0.02, P = 0.815
Total work (kJ/kg)					
AGC	1.24 ± 0.20	1.22 ± 0.20	1.31 ± 0.22	Time: d = –0.14, P < 0.001∗	Time: d = 0.36, P < 0.001∗
Control	1.24 ± 0.20	1.21 ± 0.19	1.31 ± 0.20	T × T: d = 0.04, P = 0.720	T × T: d = –0.04, P = 0.737
$\dot{V}$O_2peak_ (L.min^-1^)					
AGC	4.22 ± 0.52	4.25 ± 0.50	4.30 ± 0.52	Time: d = 0.16, P = 0.139	Time: d = 0.20, P < 0.001∗
Control	4.21 ± 0.53	4.14 ± 0.51	4.31 ± 0.51	T × T: d = 0.26, P = 0.014∗	T × T: d = –0.01, P = 0.907
$\dot{V}$O_2peak_ (mL.kg.min^–1^)					
AGC	54.1 ± 7.2	54.6 ± 6.9	55.3 ± 7.3	Time: d = 0.13, P = 0.222	Time: d = 0.18, P < 0.001∗
Control	54.5 ± 7.1	53.8 ± 6.8	56.1 ± 6.9	T × T: d = 0.21, P = 0.044∗	T × T: d = –0.10, P = 0.324
Max HR (bpm)					
AGC	179 ± 12	172 ± 12	179 ± 12	Time: d = –0.72, P < 0.001∗	Time: d = 0.03, P = 0.547
Control	178 ± 9	171 ± 10	180 ± 10	T × T: d = 0.03, P = 0.782	T × T: d = –0.07, P = 0.491

Sustained exertion test					

$\dot{V}$O_2_ (L.min^–1^)					
AGC	3.19 ± 0.34	3.25 ± 0.34	3.17 ± 0.39	Time: d = 0.08, P = 0.063	Time: d = 0.05, P = 0.669
Control	3.21 ± 0.35	3.20 ± 0.39	3.14 ± 0.37	T × T: d = 0.46, P < 0.001∗	T × T: d = 0.38, P < 0.001∗
$\dot{V}$O_2_ (mL.kg.min^–1^)					
AGC	41.0 ± 4.8	41.8 ± 4.7	40.6 ± 5.1	Time: d = 0.09, P = 0.039∗	Time: d = –0.13, P = 0.004∗
Control	41.5 ± 4.4	41.5 ± 4.9	40.7 ± 4.8	T × T: d = 0.40, P < 0.001∗	T × T: d = 0.20, P = 0.054
RER					
AGC	0.91 ± 0.03	0.86 ± 0.03	0.90 ± 0.03	Time: d = –1.39, P < 0.001∗	Time: d = –0.16, P = 0.118
Control	0.90 ± 0.03	0.88 ± 0.03	0.90 ± 0.02	T × T: d = –0.61, P < 0.001∗	T × T: d = –0.23, P = 0.031∗
Gross efficiency (%)					
AGC	21.4 ± 1.8	21.2 ± 1.3	21.6 ± 1.3	Time: d = 0.00, P = 0.992	Time: d = 0.24, P = 0.003∗
Control	21.4 ± 1.5	21.5 ± 1.5	21.9 ± 1.4	T × T: d = –0.37, P < 0.001∗	T × T: d = –0.33, P = 0.002∗
HR (bpm)					
AGC	157 ± 12	152 ± 12	154 ± 12	Time: d = –0.54, P < 0.001∗	Time: d = –0.40, P < 0.001∗
Control	157 ± 12	151 ± 11	154 ± 11	T × T: d = 0.11, P = 0.287	T × T: d = 0.09, P = 0.367
RPE					
AGC	15.1 ± 1.5	16.3 ± 1.7	14.6 ± 1.5	Time: d = 0.88, P < 0.001∗	Time: d = –0.14, P = 0.196
Control	14.8 ± 1.4	16.1 ± 1.6	14.2 ± 1.5	T × T: d = –0.09, P = 0.397	T × T: d = 0.09, P = 0.381

Abbreviations: AGC, almonds, dried grapes, and cranberries; *d*, Cohen’s *d* (i.e., standardized mean difference); HR, heart rate; HT, heavy training; LT, light training; RER, respiratory exchange ratio; RPE, rating of perceived exertion; T, taper training; T × T, Treatment × Time interaction for AGC compared to control; $\dot{V}$O2, volume of oxygen.

∗*P* < 0.05.

From LT to HT, both relative and absolute volume of peak oxygen uptake increased in AGC compared to CON, but both were increased by T compared with LT, with no difference between treatments. Maximum HR decreased from LT to HT but was no longer different from LT by T.

Relative 5CTT performance was positively associated with plasma F_2_-Isop (β = 0.0001, *P* = 0.02) and urinary nitrite concentrations (β = 0.0002, *P* = 0.01), but there were no associations with plasma nitrite (β = –0.0000008, *P* = 0.75), urinary F_2_-Isop (β = –0.003, *P* = 0.79), CK (β = –0.0001, *P* = 0.08), plasma (β = –0.0003, *P* = 0.58) or urinary (β = –0.00002, *P* = 0.42) nitrate.

### SET

Physiological parameters during the SET (45 mins of cycling at 70% maximal aerobic power output) are provided in Table 4. There were no differences in prescribed power output between groups (AGC: 235 ± 30 W; CON: 236 ± 31 W; *P* = 0.84). Across the exercise time periods (10–15 min, 25–30 min, and 40–45 min), there were increases in the absolute and relative volume of oxygen, HR, and RPE and decreases in respiratory exchange ratio (RER) and gross efficiency. However, no 3-way interactions were evident between treatment, timepoint of assessment (i.e., LT, HT, or T), and exercise time period; thus, data for each separate exercise time period has been combined in Table 4.

Compared with LT, absolute and relative oxygen uptake were maintained in AGC and reduced slightly in CON across HT and T, which resulted in a small decrease in gross mechanical efficiency in AGC. Concomitantly, RER was reduced at HT (*d* = –0.61, *P* < 0.001) and T (*d* = –0.23, *P* = 0.031) in AGC compared with CON. There was also a decrease in HR at both HT and T compared with LT and an increase in RPE at HT, but there was no difference between treatments.

### Subjective recovery

**FIGURE 2:**
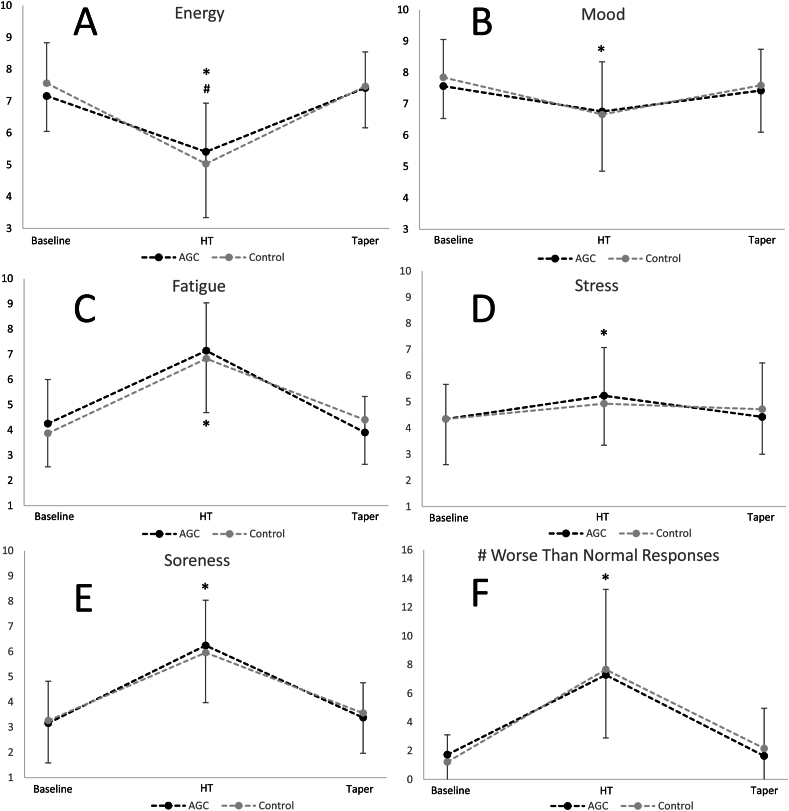
Subjective wellbeing scores. (A) Changes in energy concentrations across timepoints; (B) Changes in mood across timepoints; (C) Changes in fatigue across timepoints; (D) Changes in stress across timepoints; (E) Changes in muscle soreness across timepoints; (F) Changes in DALDA “worse than normal responses” across timepoints. Y-axes for energy, mood, fatigue, stress, and soreness indicate visual analog rating scores (out of 10), and the y-axis for # of worse than normal responses indicates the number of “worse than normal responses” in parts (A) and (B) of the DALDA. AGC, almonds, dried grapes, and cranberries; DALDA, daily analysis of life demands for athletes; HT, heavy training. ∗*P* < 0.05 compared to baseline (time effect). ^#^*P* < 0.05 compared to control (interaction effect).

All subjective wellbeing scores were worse by the end of HT compared with LT (*P* ≤ 0.006; Figure 2). Moderate to large declines were exhibited for mood (*d* = –0.71) and energy (*d* = –1.44), along with small to large increases in stress (*d* = 0.41), fatigue (*d* = 1.69), soreness (*d* = 1.71) and the number of “worse than normal” wellbeing responses (*d* = 1.75). Wellbeing scores were not different from LT by T. However, AGC maintained higher energy concentrations at HT (*d* = 0.21; *P* = 0.02) and reported experiencing less fatigue (*d* = –0.20; *P* = 0.05) at T.

## Discussion

This study demonstrated that compared with CON, consumption of AGC had no effects on limiting the physical overreaching response to 2 wk of intensified (heavy) training, nor any effect on improvements in endurance exercise performance following a subsequent 2-wk taper. Consumption of these foods did, however, improve some subjective feelings of recovery (energy) and NO bioavailability. In addition, during submaximal steady-state exercise, AGC increased oxygen consumption and fat oxidation (i.e., lower RER) and reduced mechanical efficiency.

Contrary to the proposed hypothesis, AGC consumption did not improve 5CTT performance at HT or T compared with CON. Although Yi et al. [18] observed increased time-trial performance (*P* = 0.053) with almond consumption, this was a within-group comparison rather than a comparison to a CON group. Other studies of almond consumption with prepost designs [35] and limited sample sizes [36] have demonstrated mixed results effectiveness regarding improving performance in time-to-exhaustion tests. Pistachios and peanuts have higher total polyphenol content than almonds but limited α-tocopherol content [37,38], with pistachio consumption resulting in impairments in time-trial performance in cyclists [39], whereas peanut consumption has been linked to improvements in exercise performance after a preload in nonathletes [40]. However, the HR and power output responses in the latter study did not appear to reflect a maximal test, and any improvement could be easily attributable to a carbohydrate-sparing effect as the CON condition consumed water only. Thus, there is currently limited evidence to support the consumption of almonds or nuts of any type to enhance endurance performance.

Previous studies of grape- and cranberry-based products have also demonstrated mixed effects on endurance exercise performance. Chronic grape juice consumption, without an acute dose, has demonstrated limited effects over CON treatments [41,42], whereas beneficial effects of acute grape juice consumption have been observed more recently [43,44]. Specific to the present trial, 2 studies found no effect of acute dried consumption on time-trial performance in trained cyclists [45,46] despite their increased concentrations of polyphenols compared with fresh grapes [47,48]. However, randomized controlled trials have shown no effects on time-trial performance in athletes following acute consumption of grape and cranberry extract [49], cranberry extract [50], or cranberry powder [51].

Although Yi et al. [18] found a within-group decrease in oxygen consumption and fat oxidation following 4 wk of almond consumption, the present study demonstrated increases in oxygen consumption and fat oxidation with AGC consumption at HT and T compared to CON, which translated to decreased gross mechanical efficiency. Increased submaximal oxygen consumption (*P* = 0.085) was also observed by Esquius et al. [36] following acute almond consumption, who also noted increased submaximal RER and HR, suggesting a likely decrease in exercise efficiency. Although polyphenol consumption has been theoretically linked to increased fat oxidation [52], human studies have indicated inconsistent results [53], including no effect within active populations following consumption of raisins [46] or a grape and apple extract [54]. Alternatively, the increase in fat oxidation (and thereby increase in oxygen consumption) could be attributed to the increase in total fat intake with AGC, as high-fat diets can cause increases in fat oxidation [55] as a result of increased fat availability, mobilization, and transport [56]. Notably, though, the discrepancy in the percentage of energy intake from fat between the groups (AGC: 39%, CON: ∼34%) is small compared with those seen in studies comparing the effects of high-fat compared with low-fat diets (high-fat: 71%, CON: 27%), as reviewed by Cao et al. [55]). However, in the present study, a modest increase in total fat intake with AGC was underpinned by a relatively large increase in monounsaturated and polyunsaturated fat, which are more readily oxidized and may promote greater fat utilization and energy expenditure at rest and during exercise [57,58].

It was hypothesized that the polyphenol content of AGC would increase NO bioavailability through upregulation of endothelial NO synthase activity and its antioxidant properties, preserving NO from degradation by ROS. Urinary nitrites were maintained at HT with AGC consumption compared with CON, suggesting greater NO bioavailability, but there were no differences in plasma nitrate or nitrite between treatment groups at any timepoint. Acute increases in NO synthesis in the hours following consumption of polyphenols in AGC [59] may have been better reflected in the 24-h urine sample but not in plasma after an overnight fast, which would reflect NO status acutely at that time. Additionally, acute exercise-induced decreases in circulating nitrite have been reported elsewhere [60,61]; thus, perhaps AGC consumption was able to better maintain circulating nitrite during strenuous training, which may partly explain why there was a difference at HT but not at T when the 24-h urine collection took place on a rest day.

An increase in NO bioavailability following AGC consumption contrasts with findings of other studies in which participants have consumed almonds [18,62,63]. This might be due to these previous studies analyzing nitrate and nitrite concentrations in plasma rather than urine, or it could be due to the inclusion of additional polyphenol-rich foods in the present study (i.e., cranberries and sultanas). However, consumption of grape products has produced inconsistent effects on measures of NO synthesis [[64], [65], [66]], whereas the effects of cranberry consumption have received little investigation despite their purported benefits for vascular function [67].

The training protocol used within this study successfully induced a state of functional overreaching at HT, as reflected by impairments in 5CTT performance, increased RPE, reduced maximal and submaximal HR, and impairments in all wellbeing measures. We hypothesized that inducing overreaching would increase oxidative stress, and although urinary F_2_-Isop increased following HT, plasma F_2_-Isop concentrations declined, with no effect of AGC consumption for either outcome. This decrease in plasma F_2_-Isop is in contrast to previous interventions that found no changes in plasma F_2_-Isop during 3 d of intensified training (2.5 h/d) via both cycling [68] and running [69]. Although both plasma and urinary F_2_-Isop normally exhibit acute increases after exercise [70], Galassetti et al. [71] found decreased serum F_2_-Isop following 7 d of high-volume (3 h/d) aerobic training, which may reflect training-induced increases in antioxidant defenses [72].

It was also hypothesized that reduced oxidative stress would attenuate exercise-induced muscle damage; however, CK was increased in AGC compared to CON at T. As a rest day preceded the testing session at T, combined with the reduced training load during T and minimal eccentric muscle contractions involved in cycling, the reason for the increased CK concentrations is unclear. A recent meta-analysis by Rickards et al. [73] found that polyphenol consumption did not attenuate the postexercise increase in CK, and although the included studies were comprised primarily of more eccentric-type exercise, similar effects have been replicated in cycling studies following polyphenol consumption [[74], [75], [76], [77]]. Consumption of pistachios or almonds specifically did not reduce markers of oxidative stress, muscle damage, and inflammation acutely following cycling [39] and downhill running [[78], [79], [80]]. These studies did report improvements in muscle soreness, but given the lack of participant blinding and/or benefits for physiological recovery, this may be attributable to a placebo effect.

The present study was subject to several limitations. Like all whole-food interventions, participants could not be blinded to the foods they were consuming; therefore, a placebo effect may have influenced time-trial performance and particularly subjectively reported measures. Further, the polyphenol content of AGC was estimated from available literature rather than being measured directly, so it cannot be ruled out that the dose of polyphenols may have been inadequate to elicit any physiological effects. Similarly, the polyphenol content of the CON (oat bars) was not directly measured, and their polyphenol content might have been higher than anticipated, which would have limited the differential in polyphenol content between AGC and CON. Additionally, although nitrate and nitrite in plasma and urine were analyzed as measures of NO bioavailability, the bioavailability of polyphenols and L-arginine were not measured, which makes it difficult to evaluate the effectiveness of their uptake from AGC. Regarding the training that participants performed, although no external measures of training load were recorded, HR was recorded during all training sessions, and there was equivalent compliance with prescribed HR training zones. Participants were also not required to restrict their background intakes of nitrate- or polyphenol-rich foods, introducing potential between-subject variability in polyphenol consumption. However, restriction of background polyphenol consumption does not appear to influence effects on exercise performance [10], and this is advantageous for ecological validity as restriction of these compounds has been shown to decrease nitrate concentrations [81] and increase exercise-induced oxidative stress [82]. Finally, although females have demonstrated no benefits for endurance exercise performance from the consumption of nitrate- or polyphenol-rich foods [10], there remains a lack of research into the responses of these interventions in females, particularly regarding nut consumption [40].

In conclusion, despite AGC consumption providing some minor benefits for subjective feelings of energy and NO bioavailability, it did not improve endurance exercise performance in healthy, recreationally trained male athletes. Although a recent meta-analysis indicated that polyphenol consumption broadly may confer a small ergogenic effect on endurance performance [10], this study provides further evidence that AGC, either on their own or in combination, are not an effective source. Additionally, potentially because of its high total fat and/or unsaturated fat content, AGC increased oxygen consumption and fat utilization and reduced gross mechanical efficiency during submaximal exercise, the implications of which are unclear in terms of potential impacts on longer-duration endurance exercise performance. Therefore, although consumption of AGC may improve NO bioavailability, this does not appear to result in any clear benefits for athletic performance or recovery.

## Author contributions

The authors’ responsibilities were as follows – NMAd, AMC, AMH, MJN, JDB: designed the research; NMAd, KC, CY: conducted data collection and analysis; NMAd, AMC, AMH, MJN, JDB: wrote the paper. JDB: had primary responsibility for the final content; and all authors: read and approved the final manuscript.

## Data availability

Data described in the manuscript will be made available upon request.

## Funding

This study was supported by the INC International Nut and Dried Fruit Council Foundation, although they were not involved in the collection, analysis, and interpretation of data or the preparation or submission of the manuscript for publication. The Almond Board of California and Mother Earth donated almonds and oat bars respectively for the study. NMAd was supported by an Australian Government Research Training Program Scholarship.

## Conflict of interest

AMC has previously provided consulting or advisory services to Nuts for Life, an Australian initiative established to provide information about the health effects of tree nuts. JDB is an editor for Current Developments in Nutrition and played no role in the journal’s evaluation of the manuscript. All other authors report no conflicts of interest.
